# Effect of dual-task exercises on balance, risk for fall and activities of daily living dependency of patients with stroke: a quasi-experimental study

**DOI:** 10.3389/fresc.2026.1709213

**Published:** 2026-01-20

**Authors:** Mona Mahmoud Ali, Dalia Abdallah Abdelatief, Mona Mohamed Saad, Heba Abdelgawad Elfeky

**Affiliations:** 1 Department of Medical Surgical Nursing, Faculty of Nursing, Ain Shams University, Cairo, Egypt; 2 Physical Medicine, Rheumatology and Rehabilitation Department, Faculty of Medicine, Ain Shams University, Cairo, Egypt

**Keywords:** activities of daily living, balance, dual-task exercises, fall risk, stroke rehabilitation

## Abstract

**Background:**

Stroke survivors often experience impaired balance, increased fall risk, and dependency in activities of daily living (ADLs). Dual-task exercises, combining motor and cognitive challenges, may improve these outcomes. This study was conducted to evaluate the effects of dual-task exercise intervention on balance, fall risk, and ADLs dependency in stroke patients.

**Methods:**

A quasi-experimental single-group pretest–posttest design was used with 54 stroke patients recruited from Ain Shams University Hospitals, Cairo. Participants underwent 16- sessions of individualized dual-task exercises over 8 weeks. Follow-up assessments were conducted immediately post-intervention (two months). Balance was measured at follow-up using the Postural Assessment Scale for Stroke (PASS); fall risk was assessed at follow-up using the Timed Up and Go Test (TUGT); and ADLs dependency was evaluated at follow-up using the Barthel Index (BI). Data were analyzed using SPSS version 27.

**Results:**

Before intervention, 75.9% of participants had low ability to maintain posture, which improved post-intervention to 61.1% with high ability (*p* < 0.05). Similarly, the ability to change posture improved significantly (81.5% low to 63.0% high; *p* < 0.05). Pre-intervention 68.5% of patients were at high risk of falling, which decreased post-intervention, with 40.7% classified as low risk and 46.3% as no risk (*p* < 0.001). Severe ADLs dependency reduced markedly from 75.9% to 9.3% (*p* < 0.001). Across time points, higher PASS scores (better balance) were negatively associated with fall risk (TUGT) and ADL dependence, while higher BI scores (better ADL independence) were negatively correlated with fall risk and positively correlated with PASS (*p* = 0.001 for all).

**Conclusion:**

Dual-task exercise training significantly improves balance, reduces fall risk, and enhances independence in ADLs among stroke patients. These findings support integrating dual-task interventions into stroke rehabilitation protocols, especially in resource-limited settings.

## Introduction

Stroke, a major neurological disorder, results from either an interruption of blood flow to the brain (ischemic stroke) or bleeding within the brain (hemorrhagic stroke). Both types cause damage to specific areas of the brain, leading to a wide range of impairments, including motor deficits, sensory disturbances, cognitive dysfunctions, and language disorders (1). Globally, stroke is recognized as the second most common cause of death and is one of the leading contributors to long-term disability (2). Over the years, the incidence and prevalence of stroke have continued to rise, resulting in substantial health burdens, with significant rates of morbidity and mortality (3).

Depending on the location and extent of brain damage, stroke survivors may experience a broad array of physical and cognitive impairments. These may include altered consciousness, sensory loss, muscle weakness, sphincter dysfunction, and impaired coordination and gait. Impairments in postural control and balance are especially critical as they are closely linked with mobility limitations and dependency in performing daily activities. Post-stroke imbalance can result from damage to either sensory or motor pathways and is a primary reason for patients becoming dependent in activities of daily living (ADLs). These functional limitations often persist long after the initial stroke event, diminishing the individual's independence and quality of life (4). Although advances have been made in stroke prevention and management, the condition remains difficult to fully prevent or treat. Stroke prevention generally focuses on managing risk factors across populations, while treatment targets the underlying pathophysiology. However, despite significant research efforts, there is still no universal method for completely preventing or reversing stroke-related damage (5).

In Egypt, the burden of stroke is particularly notable, with an estimated incidence rate of 240 per 100,000 people, translating to about 250,000 new cases annually. Of these, approximately 10% succumb within the first month, and many others are left with various levels of disability (6). The post-stroke period is often characterized by challenges such as reduced mobility, poor balance, and deficits in memory, perception, attention, and emotional regulation (7). Consequently, stroke rehabilitation is essential for restoring function and promoting recovery. It aims to address common sequelae such as muscle weakness, altered muscle tone, poor coordination, proprioceptive dysfunction, and gait disturbances factors that contribute significantly to ADL dependency (8).

Balance, defined as the ability to maintain postural stability by adjusting the body's position relative to its center of gravity, is vital for regaining independence in stroke survivors (9, 10). ADLs, which include fundamental tasks such as dressing, grooming, mobility, and transferring, are often compromised following a stroke. Effective performance of these tasks depends on a combination of motor and cognitive functions, especially during movement and balance-related actions (11). A study has shown that approximately 35% of stroke survivors remain dependent on others for basic ADLs even one-year post-stroke (12). Impaired balance is a key contributor to this dependency, limiting safe mobility and increasing fall risk (13). Given these challenges, rehabilitation strategies that can simultaneously improve balance, reduce fall risk, and enhance ADL independence are of critical importance. The current study explores the use of dual-task exercises as an intervention in stroke rehabilitation at Ain Shams University Hospitals in Cairo, Egypt. The aim is to evaluate their effect on improving balance, decreasing the risk of falls, and promoting greater independence in ADLs among stroke patients.

## Materials and methods

### Study design

This study adopted a quasi-experimental approach, using a single-group pretest-posttest design. In this method, participants were evaluated both before and after receiving the dual-task exercise intervention to determine its effects on balance, fall risk, and dependency in activities of ADLs among patients recovering from stroke.

### Study setting

The study was conducted at the outpatient clinic of the Physical Medicine, Rheumatology, and Rehabilitation Department at Ain Shams University Hospitals in Cairo, Egypt; a major tertiary care facility. The clinic is well-equipped with specialized rehabilitation services, including physiotherapy and occupational therapy units, and serves stroke patients. It provided a suitable environment with trained professionals, proper equipment, and space for safely implementing and monitoring the 16-session dual-task exercise intervention.

### Study period

The study was conducted over a period of six months, starting from September 2024 and concluding at the end of February 2025. During this time, participant recruitment, baseline assessments (pretests), the 16-session dual-task exercise intervention, and post-intervention evaluations (posttests) were completed. This timeframe allowed for consistent follow-up and comprehensive monitoring of participants' progress throughout the study.

### Study population

The study population consisted of adult stroke patients attending the outpatient clinic at the Physical Medicine, Rheumatology, and Rehabilitation Department of Ain Shams University Hospitals in Cairo, Egypt. These patients were receiving follow-up care and rehabilitation services following a recent stroke.

The inclusion criteria for this study required participants to be adult patients of either gender who had been recently diagnosed with stroke (within six months), were medically stable, able to walk independently or with the use of assistive devices, willing to participate, and capable of following instructions related to the dual-task training program. Patients were excluded if they had unstable medical conditions; additional musculoskeletal or neurological disorders that significantly impaired balance (such as cerebellar or basal ganglia disorders or severe joint diseases); major cognitive impairment (e.g., Alzheimer's disease or dementia) was explicitly excluded to ensure informed consent could be reliably obtained and to maintain consistent engagement with the training and assessments. This aligns with ethical standards and supports protocol adherence.

### Sample size and sampling technique

The sample size for this study was calculated using a statistical formula based on the total number of stroke patients treated at Ain Shams University Hospitals in 2023 (*N* = 120), with a confidence level of 95% (z = 1.96), an acceptable margin of error of 10% (d = 0.10), and an estimated population proportion of 0.50 (P). This calculation determined that a sample of 54 participants was sufficient for the study. A purposive sampling technique was used to select these participants, ensuring that individuals who met the study's specific inclusion and exclusion criteria were chosen to participate. This approach allowed for the targeted recruitment of stroke patients suitable for the dual-task exercise intervention. There were no dropouts in this study, as all participants completed the intervention until the end. Potential confounding factors, such as the stage of stroke, were controlled by carefully applying the inclusion and exclusion criteria.

### Data collection

Data were collected using four main instruments as follows:

#### Part I: A structured patient interview questionnaire

This questionnaire was a structured patient interview questionnaire, developed by the investigator in English based on a review of related literature. This questionnaire gathered demographic data, including age, gender, marital status, educational level, occupation, and residence, through six multiple-choice questions designed to capture essential patient characteristics.

#### Part II: postural assessment scale for stroke (PASS)

In the current study, the instrument was adapted and translated into Arabic for this study. PASS evaluates post-stroke balance through 12 items divided into two sections: maintaining posture and changing posture. The maintaining posture section includes tasks such as sitting without support and standing on the paretic or nonparetic leg, while the changing posture section involves movements like shifting from supine to sitting and standing up. Each item is scored from 0 to 3, with a maximum total score of 36, which is then converted into a percentage to classify balance levels as high, moderate, or low (14).

#### Part III: timed up and go test (TUGT)

The third tool was the Timed Up and Go Test (TUGT), also adapted and translated into Arabic. This test measures functional mobility and fall risk by timing the patient as they rise from a chair, walk a short distance, turn around, return, and sit down. The time taken is recorded in seconds and categorized into normal mobility, low, moderate, or high fall risk based on established cutoff points (15).

#### Part IV: Barthel index (BI) scale

Finally, the Barthel Index (BI) Scale was used to assess the level of dependency in ADLs. Adapted and translated into Arabic, this scale evaluates 10 items such as bowel and bladder control, grooming, feeding, transfers, mobility, dressing, and bathing. Each activity is scored from 0 (unable) to 2 (independent), with a total raw score of 20 multiplied by 5 to yield a score out of 100. Scores are then classified to reflect levels of dependency, ranging from slight to total dependency (16).

### Procedure and intervention

Patients were approached during their visits to the outpatient clinic of the Physical Medicine, Rheumatology, and Rehabilitation Department at Ain Shams University Hospitals, Cairo, Egypt. The study's purpose and the nature of the dual-task exercises were clearly explained to them. All participants were assigned to a single group, and baseline measurements were collected using the pre-designed study tools after initial assessment. The dual-task exercise program was then tailored and initiated for each patient.

Participants completed a total of 16 sessions of dual-task exercises over eight weeks, with two sessions per week. Each session lasted approximately 30 min, and training was individualized to meet each patient's needs (9). The exercises were conducted under the supervision of a physical therapist, with the investigator leading the sessions. Exercises progressed through different challenge levels, with each level lasting two weeks. The intensity and difficulty of tasks increased gradually based on patients' feedback and their physical responses. The final exercise level varied for each patient depending on their tolerance and progress.

The duration and frequency of the intervention were based on previous research by Pang et al. (9), which demonstrated that 8 weeks of dual-task exercises, 30 min per session, effectively improved balance. The investigator was responsible for both data collection and conducting the intervention. The 16 sessions were structured as follows: the first four sessions focused on single-task balance training, including activities such as dynamic weight shifting, sit-to-stand exercises, and seated heel raises.

The next four sessions involved cognitive-motor dual-task training, such as walking while narrating stories, giving opposite directional commands, and sitting while reciting numbers, days, or months backward. The following four sessions emphasized motor-motor dual-task balance exercises, including walking while kicking a ball, standing while throwing and catching a ball, and walking while carrying a cup of water without spilling it. The final four sessions simulated everyday activities, such as transitioning from sitting to standing and walking, or sitting to standing and picking up objects from the floor.

The initial four sessions establish safe, efficient single-task balance control to minimize risk and ensure participant readiness for heavier cognitive motor demands. The subsequent four sessions introduce cognitive motor dual-task requirements, followed by motor–motor dual-task balance challenges, and finally functional/ADL tasks within a dual-task context. This progression aligns with safety considerations and rehabilitation principles, ensuring gradual loading of dual-task demands while preserving task specificity and functional relevance. Throughout, the program advances from foundational balance training to progressively complex dual-task and functional activities, with the understanding that substantial dual-task demands are most effectively trained after establishing baseline motor control and safety.

#### Content validity and reliability

The validity of the face and content of the study instruments, the questionnaire were determined by a panel of five experts in nursing administration, two professors from the department of medical surgical nursing, Ain Shams University. Three professors from the department of medical surgical nursing at Cairo University. The experts revised the tools for clarity, relevance, completeness, easy language, and usability; Some changes were made while the final forms were developed. Since the original instruments were developed in English, cultural and linguistic adaptation was performed to ensure appropriateness for the Egyptian healthcare context. In addition, the reliability of the tools developed was tested using Cronbach's alpha coefficients, the PASS internal consistency with Cronbach's alpha 0.89. The BI Scale of ADLs internal consistency with Cronbach's alpha 0.90. The ABC scale internal consistency with Cronbach's alpha 0.82. In addition, Testing reliability of the Timed Up and Go Test (TUGT) tool through test-retest reliability analysis Intraclass Correlation Coefficient (ICC) is 0.93.

#### Pilot study

A pilot study was carried out in September 2024, and was carried out in 10% of the sample size (6 patients) who participated in a pilot study to evaluate the applicability, clarity, and effectiveness of the tools and to estimate the time to fill it, which ranged between about 20–30 min. No adjustments or changes were made and patients were added to the findings of the pilot study findings.

### Ethics statement

This study was conducted in accordance with the ethical principles of the Declaration of Helsinki and its later amendments. A formal approval was obtained from the Research Ethic Committee of the Faculty of Nursing, Ain Shams University, Egypt. Registered number 25.08.832, to carry out the study. Additionally, participants were thoroughly informed about the study's purpose and procedures, and written informed consent was obtained from all. It was clearly communicated that participation was voluntary, and all collected data would be kept strictly confidential. participants have the right to withdraw from the study without any reason at any time.

### Data analysis

The data were analyzed using SPSS version 27 and Excel 2016. Quantitative results were summarized as means, standard deviations, and percentages. The Marginal Homogeneity test assessed differences between pre- and post-intervention scores for balance (PASS), fall risk, and ADL dependency. Spearman's correlation coefficient measured the relationships between fall risk, balance, and ADL levels. A *p*-value < 0.05 was considered statistically significant.

## Results

A total of 54 stroke patients were enrolled in the study, including those who participated in the pilot phase, as no changes were made to the study protocol afterward. According to Table 1, the majority of participants (64.8%) were aged between 45 and less than 60 years, with a mean age of 53.49 ± 9.94 years. Most were female (66.7%) and married (94.4%). Regarding education, (40.7%) could read and write, (55.6%) were employed. Notably, (53.7%) resided in rural areas, while (46.3%) were urban dwellers. Most participants were in the chronic stage of stroke (68.5%). The predominance of rural residence and high marriage rate among participants may reflect stronger caregiver support and potential transportation barriers to clinic-based therapy. which may have contributed positively to rehabilitation outcomes due to strong familial support.

**Table 1:** Demographic characteristics of the study participants.

Variables	*N* (%)
Age (in years)	
18- < 30	2 (3.7)
30- < 45	4 (7.4)
45- < 60	35 (64.8)
>60	13 (24.1)
Mean ± SD	53.49 ± 9.94
Gender	
Male	18 (33.3)
Female	36 (66.7)
Marital status	
Not married	3 (5.6)
Married	51 (94.4)
Education level	
Read and write	22 (40.7)
Primary school	7 (13.0)
Secondary school	16 (29.6)
University	6 (16.7)
Occupation	
Employed	30 (55.6)
Unemployed	24 (44.4)
If you employed, mention what is the type of work (*n* = 30)	
It requires muscular effort	17 (56.6)
Office work	13 (43.3)
Residence	
Rural	29 (53.7)
Urban	25 (46.3)
Stroke stage	
Acute	17 (31.5)
Chronic	37 (68.5)

N, number;(%), Percentage; SD, standard deviation.

Table 2 shows that, there was a significant statistical difference between mean score of pre and post implementation dual tasks exercises regarding all items of balance confidence in performance of activities at *P*-value ≤ 0.05.

**Table 2:** Comparison between pre/post implementation of dual tasks exercises among patients with stroke regarding balance confidence in performance of activities without losing balance activities.

Activities	Pre		Post		Paired *t*-test	
	Mean	SD	Mean	SD	*t*-test	*p*-value
Walk around the house	28.52	15.711	48.33	20.258	9.482	˂0.001*
Walk up or down stairs	31.67	16.795	52.41	17.368	10.792	˂0.001*
Bend over and pick up a slipper from front of a closet floor	33.52	18.031	52.59	19.346	8.317	˂0.001*
Reach for a small can off a shelf at eye level	33.33	16.711	54.63	18.193	8.840	˂0.001*
Stand on tip toes and reach for something above your head	33.15	17.137	54.26	18.388	9.433	˂0.001*
Stand on a chair and reach for something	32.96	17.335	52.78	20.227	8.627	˂0.001*
Sweep the floor	31.67	15.631	55.37	20.532	9.597	˂0.001*
Walk outside the house to a car parked in the driveway	35.37	18.297	56.11	19.563	9.743	˂0.001*
Get into or out of a car	35.74	18.794	53.52	21.382	7.224	˂0.001*
Walk across a parking lot to the mall	32.78	16.758	53.52	19.246	9.388	˂0.001*
Walk up or down a ramp	29.26	17.682	50.56	19.173	11.276	˂0.001*
Walk in a crowded mall where people rapidly walk past you	33.70	15.817	53.15	18.411	7.959	˂0.001*
Runs into people while they are walking in the mall	33.89	17.311	55.19	19.107	10.574	˂0.001*
Climbs into and out of the escalator while holding the railings	29.81	19.279	53.89	20.596	11.083	˂0.001*
Climbs into or out of the escalator while holding their personal belongings, so they can't hold the handrail	29.81	20.234	51.48	19.658	10.347	˂0.001*
Walk outside on sliding sidewalks	29.26	17.894	49.81	19.473	10.174	˂0.001*
Total	515.44	245.33	847.59	278.55	12.315	˂0.001*

*p*-value > 0.05 = Non-significant (NS);*p-value ≤ 0.05 = Significant (S).

As shown in Table 3, prior to the intervention, 75.9% of patients had a low ability to maintain posture, which improved post-intervention, with 61.1% demonstrating a high ability. Similarly, 81.5% had a low ability to change posture before the exercises, improving to 63.0% showing a high ability after the intervention. A statistically significant improvement was found in all PASS balance domains before and after the dual-task exercise program (*p* < 0.05).

**Table 3:** Comparison between pre/post implementation of dual-task exercises among patients with stroke regarding total level of PASS domain.

Domains of balance	Pre		Post		MH test	
	*N*	%	*N*	%	Test	*p*-value
Maintaining a posture						
Low	41	75.9	16	29.6	37.532	˂0.001*
Moderate	10	18.5	5	9.3		
High	3	5.6	33	61.1		
Changing a posture						
Low	44	81.5	16	29.6	34.727	˂0.001*
Moderate	5	9.3	4	7.4		
High	5	9.3	34	63.0		
Total level of Postural Assessment Scale for Stroke (PASS)						
Low	44	81.5	13	24.1	39.300	˂0.001*
Moderate	7	13.0	12	22.2		
High	3	5.6	29	53.7		

MH test = Marginal Homogeneity Test; *p-value < 0.05 = Statistically significant.

Table 4 presents pre- and post-implementation differences in fall risk categories (from TUG-based assessment) and BI-defined ADL dependency. Table 4 reveals that 68.5% of patients were at high risk of falling before the intervention, which decreased post-intervention, with 40.7% and 46.3% of patients categorized as low risk and no risk, respectively. This improvement was statistically significant (*p* < 0.001). Additionally, severe ADL dependency was observed in 75.9% of participants before the intervention, which dropped significantly to 9.3% afterward, also showing a highly significant difference (*p* < 0.001).

**Table 4:** Comparison between pre/post implementation of dual-task exercises among the studied patients regarding risk of falling and total level of dependency in ADL.

Risk of falling	Pre		Post		MH test	
	*N*	%	*N*	%	Test	*p*-value
Normal	0	0.0	25	46.3	42.412	˂0.001*
Low risk of falling	3	5.6	22	40.7		
Moderate risk of falling	14	25.9	6	11.1		
High risk of falling	37	68.5	1	1.9		
Total level of dependency in ADLs	Pre		Post		MH test	
	N	%	N	%	Test	*p*-value
Total dependency	6	11.1	1	1.9	18.454	˂0.001*
Severe dependency	41	75.9	5	9.3		
Moderate dependency	7	13.0	27	50.0		
Slight dependency	0	0.0	21	38.9		

MH test = Marginal Homogeneity Test; *p-value < 0.05 = Statistically significant.

Table 5 indicates a significant negative correlation between fall risk (TUG-based), BI-defined ADL dependency levels, and PASS balance scores, both pre- and post-intervention (*p* = 0.001). In other words, higher fall risk and greater ADL dependency are associated with poorer balance as measured by PASS, before and after the intervention.

**Table 5:** Correlation matrix between the studied variable among of patients with stroke pre and post implementation dual- task exercises.

Total level		Balance PASS		Risk of falling	
		r	*p*-value	r	*p*-value
Risk of falling	Pre	−0.777	0.001*	–	–
	Post	−0.765	0.001*	–	–
Level of ADL	Pre	0.730	0.001*	−0.610	0.001*
	Post	0.683	0.001*	−0.684	0.001*

R = Spearman correlation coefficient; *p-value < 0.05 = Statistically significant.

Conversely, there was a significant positive correlation between PASS balance scores and ADL performance both pre- and post-intervention (*p* = 0.001). This indicates that better balance is associated with greater independence in ADLs across the study period.

## Discussion

This study provides robust evidence that dual-task exercises significantly improve balance, reduce fall risk, and enhance independence in ADLs among stroke patients. The intervention supported by a simple, illustrated booklet and video-guided exercises, proved effective, especially among a population characterized by diverse educational, occupational, and socioeconomic backgrounds. These findings are consistent with a growing body of evidence emphasizing the effectiveness of task-specific and cognitively engaging rehabilitation approaches in stroke recovery. The observed improvements in balance ability are in line with prior studies. Zhou et al. (16) reported that dual-task training leads to significant gains in balance performance among individuals with chronic stroke. Similarly, De Luca et al. (17) demonstrated statistically significant balance improvements maintained at follow-up, affirming the long-term benefit of such interventions. Zhang et al. (18) also emphasized the superiority of dual-task training over conventional single-task methods in enhancing static balance.

Evidence suggests improvements in balance, with more variable effects on autonomy in ADLs depending on the intervention dose and task demands. For example, recent subacute work highlighting goal-oriented dual-task and proprioceptive training indicates improvements in balance and ADL autonomy, with effects on fall risk observed during dual-task performance when proprioceptive goals are emphasized Chiaramonte et al. (19). Importantly, this study emphasizes that the pivotal element may be goal-oriented proprioceptive training, with the distinction between single- vs. dual-task approaches sometimes appearing less critical than the accurate targeting of motor-cognitive goals.

Regarding fall risk, our findings align with Ahmed et al. (20), who highlighted that dual-task training contributes to fall prevention and enhances mobility. The discrepancy with Cortés-Pérez et al. (21), who reported even greater reductions in fall risk, may be attributed to methodological differences, such as intervention intensity, patient population, and duration. This study also found a notable decrease in patient dependency in ADLs following the intervention. These results support De Sousa et al. (22), who emphasized the benefit of sit-to-stand and other functional exercises in regaining independence. An & Kim (23) further supported the notion that dual-task exercises improve quality of life and daily functioning. Additionally, the significant negative correlation observed between improved balance (PASS scores) and fall risk indicates a strong inverse relationship, validating the idea that enhanced postural control leads to a safer, more stable functional state. This finding is consistent with the work of Mohammed et al. (24), who reported similar outcomes in intervention groups practicing dual-task training. Similarly, Vecchio et al. (25) indicates that proprioceptive training with dual-task components can positively influence gait parameters in chronic stroke. This supports the idea that integrating proprioceptive goals with dual-task demands can maximize walking outcomes, with potentially robust effects on gait speed. Our study's broader functional outcomes are compatible with these findings, underscoring that improvements in gait-related measures can accompany balance and ADL gains when interventions are well-structured and goal-directed.

Several factors contributed to the intervention's success. The structured design of dual-task exercises, combined with accessible educational tools (booklet and video links), facilitated patient understanding, adherence, and home-based practice. Starting with simple activities and progressively introducing more complex ones allowed for gradual skill acquisition while minimizing frustration or risk. Social and cultural dynamics also played a role. The majority of participants were married and from rural areas, which may have contributed positively to rehabilitation outcomes due to strong familial support. Moreover, the inclusion of working-age individuals underscores the importance of offering interventions suitable for individuals eager to return to daily responsibilities and employment. The study's implementation in a government hospital setting, where patients typically come from lower socioeconomic backgrounds, also demonstrates the intervention's relevance and adaptability to under-resourced environments.

Based on the study's findings, several key recommendations can be proposed for healthcare policymakers and rehabilitation planners to improve stroke rehabilitation outcomes. First, dual-task training should be formally integrated into standard stroke rehabilitation protocols, as it has been shown to significantly enhance balance, reduce fall risk, and improve independence in daily activities. Second, there is a clear need to develop and nationally distribute illustrated educational booklets, particularly in rural or low-literacy settings, to empower patients to continue exercises at home. Third, community-based rehabilitation services should be expanded to include guided dual-task sessions, especially in underserved regions where access to specialized care may be limited. Fourth, it is essential to train healthcare providers including physical therapists, nurses, and community health workers, on delivering and adapting dual-task exercises to individual patient needs. Fifth, considering that many stroke survivors are of working age, workplace health promotion programs should include stroke risk screenings and preventive strategies, such as balance-focused exercises and stress management. Lastly, to ensure long-term sustainability and effectiveness, funding should be directed toward research and follow-up programs that evaluate the extended impact of dual-task training and help shape evidence-based health policy.

### Strengths and limitations

This study has several notable strengths, including the use of a practical and accessible intervention, an easy-to-understand booklet accompanied by video guidance, which made dual-task training feasible even for participants with limited literacy. The inclusion of a diverse sample with varying ages, education levels, and residential settings enhances the generalizability of the findings. Additionally, the comprehensive assessment of balance, fall risk, and dependency in ADLs provides a well-rounded evaluation of functional recovery. However, the study is not without limitations. The absence of a control group limits the ability to attribute observed improvements solely to the intervention, while the short duration of follow-up prevents assessment of long-term outcomes. Furthermore, reliance on self-reported measures may introduce recall or observer bias, and varying levels of adherence to home exercises, potentially influenced by differences in motivation or support, could have affected individual results.

## Conclusion

This study highlights the clinical value of dual-task exercises in stroke rehabilitation, showing significant improvements in balance, fall risk reduction, and ADL independence. The use of accessible educational materials, such as booklets and video tutorials, allowed patients to practice effectively at home, supporting continued recovery beyond hospital settings. These findings are particularly relevant for low-resource and rural healthcare environments, where professional supervision may be limited. Given the rising prevalence of stroke among middle-aged working individuals, particularly women, and those from lower socioeconomic backgrounds, integrating dual-task training into national rehabilitation strategies is both necessary and urgent. Healthcare systems should adopt dual-task interventions as a low-cost, high-impact solution to improve outcomes and quality of life for stroke survivors.

## Data Availability

The original contributions presented in the study are included in the article/Supplementary Material, further inquiries can be directed to the corresponding author.

## References

[1] Shaik NF Regan RF Naik UP. Platelets as drivers of ischemia/reperfusion injury after stroke. Blood Advances. (2021) 5(5):1576–84. 10.1182/bloodadvances.2020002888 33687431 PMC7948278

[2] Pu L Wang L Zhang R Zhao T Jiang Y Han L. Projected global trends in ischemic stroke incidence, deaths and disability-adjusted life years from 2020 to 2030. Stroke. (2023) 54(5):1330–9. 10.1161/STROKEAHA.122.040073 37094034

[3] Tu W-J Wang L-D. China Stroke surveillance report 2021. Mil Med Res. (2023) 10(1):33. 10.1186/s40779-023-00463-x 37468952 PMC10355019

[4] Gittins M Lugo-Palacios D Vail A Bowen A Paley L Bray B Stroke impairment categories: a new way to classify the effects of stroke based on stroke-related impairments. Clin Rehabil. (2021) 35(3):446–58. 10.1177/0269215520966473 33131321 PMC7944424

[5] Kuriakose D Xiao Z. Pathophysiology and treatment of stroke: present status and future perspectives. Int J Mol Sci. (2020) 21(20):7609. 10.3390/ijms21207609 33076218 PMC7589849

[6] Feigin VL Stark BA Johnson CO Roth GA Bisignano C Abady GG Global, regional, and national burden of stroke and its risk factors, 1990–2019: a systematic analysis for the global burden of disease study 2019. Lancet Neurol. (2021) 20(10):795–820. 10.1016/S1474-4422(21)00252-0 34487721 PMC8443449

[7] Yao Y-Y Wei Z-J Zhang Y-C Li X Gong L Zhou J-W Functional disability after ischemic stroke: a community-based cross-sectional study in Shanghai, China. Front Neurol. (2021) 12:649088. 10.3389/fneur.2021.649088 34512499 PMC8427524

[8] Hartley T Burger M Inglis-Jassiem G. Post stroke health-related quality of life, stroke severity and function: a longitudinal cohort study. African J Disabil. (2022) 11:1–10. 10.4102/ajod.v11i0.947 PMC8831922 35169551

[9] Pang MYC Yang L Ouyang H Lam FMH Huang M Jehu DA. Dual-task exercise reduces cognitive-motor interference in walking and falls after stroke: a randomized controlled study. Stroke. (2018) 49(12):2990–8. 10.1161/STROKEAHA.118.022157 30571419

[10] Kerse N Parag V Feigin VL McNaughton H Hackett ML Bennett DA Falls after stroke: results from the auckland regional community stroke (ARCOS) study, 2002 to 2003. Stroke. (2008) 39(6):1890–3. 10.1161/STROKEAHA.107.509885 18483413

[11] Appelros P Nydevik I Terént A. Living setting and utilisation of ADL assistance one year after a stroke with special reference to gender differences. Disabil Rehabil. (2006) 28(1):43–9. 10.1080/09638280500165278 16393832

[12] Saraiva J Rosa G Fernandes S Fernandes JB. Current trends in balance rehabilitation for stroke survivors: a scoping review of experimental studies. Int J Environ Res Public Health. (2023) 20(19):6829. 10.3390/ijerph20196829 37835099 PMC10572981

[13] Benaim C Pérennou DA Villy J Rousseaux M Pelissier JY. Validation of a standardized assessment of postural control in stroke patients: the postural assessment scale for stroke patients (PASS). Stroke. (1999) 30(9):1862–8. 10.1161/01.STR.30.9.1862 10471437

[14] Beauchet O Annweiler C Assal F Bridenbaugh S Herrmann FR Kressig RW Imagined timed up & go test: a new tool to assess higher-level gait and balance disorders in older adults? J Neurol Sci. (2010) 294(1-2):102–6. 10.1016/j.jns.2010.03.021 20444477

[15] Quinn TJ Langhorne P Stott DJ. Barthel index for stroke trials: development, properties, and application. Stroke. (2011) 42(4):1146–51. 10.1161/STROKEAHA.110.598540 21372310

[16] Zhou Q Yang H Zhou Q Pan H. Effects of cognitive motor dual-task training on stroke patients: a RCT-based meta-analysis. J Clin Neurosci. (2021) 92:175–82. 10.1016/j.jocn.2021.08.009 34509248

[17] De Luca A Squeri V Barone LM Vernetti Mansin H Ricci S Pisu I Dynamic stability and trunk control improvements following robotic balance and core stability training in chronic stroke survivors: a pilot study. Front Neurol. (2020) 11:494. 10.3389/fneur.2020.00494 32625162 PMC7311757

[18] Zhang L Ma J Liu X Jin A Wang K Yin X. Cognitive-motor dual-task training on gait and balance in stroke patients: meta-analytic report and trial sequential analysis of randomized clinical trials. J Neuroeng Rehabil. (2024) 21(1):227. 10.1186/s12984-024-01507-6 39716165 PMC11665123

[19] Chiaramonte R D’Amico S Caramma S Grasso G Pirrone S Ronsisvalle MG The effectiveness of goal-oriented dual task proprioceptive training in subacute stroke: a retrospective observational study. Ann Rehabil Med. (2024) 48(1):31–41. 10.5535/arm.23086 38433007 PMC10915301

[20] Ahmed U Karimi H Amir S Ahmed A. Effects of intensive multiplanar trunk training coupled with dual-task exercises on balance, mobility, and fall risk in patients with stroke: a randomized controlled trial. J Int Med Res. (2021) 49(11):03000605211059413. 10.1177/03000605211059413 34812070 PMC8647262

[21] Cortés-Pérez I Nieto-Escamez FA Obrero-Gaitán E. Immersive virtual reality in stroke patients as a new approach for reducing postural disabilities and falls risk: a case series. Brain Sci. (2020) 10(5):296. 10.3390/brainsci10050296 32429085 PMC7287864

[22] de Sousa DG Harvey LA Dorsch S Varettas B Jamieson S Murphy A Two weeks of intensive sit-to-stand training in addition to usual care improves sit-to-stand ability in people who are unable to stand up independently after stroke: a randomised trial. J Physiother. (2019) 65(3):152–8. 10.1016/j.jphys.2019.05.007 31227279

[23] An H-S Kim D-J. Effects of activities of daily living-based dual-task training on upper extremity function, cognitive function, and quality of life in stroke patients. Osong Public Health Res Perspect. (2021) 12(5):304. 10.24171/j.phrp.2021.0177 34719222 PMC8561019

[24] Mohammed Mohammed A Ahmed Hassanein A Gaber Mohammed H Kamal Hamed M Sobhy Omran E. Effects of dual task exercises training program on stroke patients. J Nurs Sci Benha Univ. (2021) 2(2):69–87. 10.21608/jnsbu.2021.186431

[25] Vecchio M Chiaramonte R Alessandro D Buccheri E Finocchiaro P Scaturro D Do proprioceptive training strategies with dual-task exercises positively influence gait parameters in chronic stroke? A systematic review. J Rehabil Med. (2024) 56:18396. 10.2340/jrm.v56.18396 39145519 PMC11337222

